# Evaluation of drug prescribing pattern based on World Health Organization drug use indicators in dermatology outpatient department of Injibara General Hospital, Northwest Ethiopia, 2024

**DOI:** 10.3389/fmed.2024.1499974

**Published:** 2025-01-08

**Authors:** Alemu Bezabih Tegegnie, Tamiru Alene, Habtamu Nega Bogale, Workineh Tamir, Meaza Molla Sineshaw

**Affiliations:** ^1^Department of Dermatovenereology, College of Medicine and Health Sciences, Injibara University, Injibara, Ethiopia; ^2^Department of Pediatrics and Child Health Nursing, College of Medicine and Health Sciences, Injibara University, Injibara, Ethiopia; ^3^World Health Organization in Papua New Guinea, Consultant of Vaccine-Preventable Diseases and Immunization, Port Moresby, Papua New Guinea; ^4^Department of Medical Laboratory Science, College of Medicine and Health Science, Injibara University, Injibara, Ethiopia; ^5^Ethiopian Statistical Services, Bahirdar, Ethiopia

**Keywords:** prescribing indicators, dermatology, drug use, Injibara, Ethiopia

## Abstract

**Background:**

Irrational use of medicines is a problem globally that soon needs to be addressed. According to estimates from the World Health Organization, almost half of all medications were improperly prescribed. This study aimed to assess the drug prescribing patterns based on World Health Organization drug use indicators in the dermatology outpatient department of Injibara General Hospital.

**Method:**

A facility-based retrospective cross-sectional study was conducted from August 15 to August 30, 2024, with 620 patient prescriptions issued at the dermatology outpatient department of Injibara General Hospital. All patient prescriptions dispensed from the dermatology outpatient department from April to July 2024 were included. A structured data collection tool adopted from the World Health Organization core medicine use indicator was used to collect data, and Statistical Package for Social Science version 27.1 was used for data analysis.

**Results:**

An average of 1.74 drugs per encounters was prescribed, with 21.6 and 3.1% of prescriptions being antibiotics and injections, respectively. Generics were used in 95.4% of prescriptions, and nearly 84% of drugs were prescribed from the Ethiopian essential-drug list.

**Conclusion:**

The World Health Organization’s recommended threshold for the average number of prescriptions prescribed per encounter was met, indicating proper prescribing practices that reduce polypharmacy. The percentage of encounters with antibiotics was within the World Health Organization’s value, which reflects that dermatologists are less likely engaging in irrational antibiotic prescriptions. Likewise, the World Health Organization’s recommendations for the percentage of encounters with injection was met, indicating an effort to minimize unnecessary use of injections by dermatologists, which can reduce complications associated with injection use. However, the World Health Organization’s guidelines for generic drug prescriptions were not met, suggesting that dermatologists are less likely to prescribe generic drugs, which can raise patient healthcare expenditures considerably. Prescriptions from the Ethiopian essential medicine list also fell short of World Health Organization’s standards, indicating a failure to follow established guidelines.

## Introduction

The biggest organ in the human body, the skin, is a component of the integumentary system (1). In Ethiopia, 28% of adults and 25% of children have one or more skin diseases, making dermatological disorders one of the main reasons for hospital visits (2). The most common dermatological problems in Ethiopia are dermatitis, pyoderma, and fungal skin infections, among others (3). Most skin diseases are chronic and require lifetime treatment, requiring appropriate diagnosis and rational drug prescription by physicians (4).

The issue of irrational use of medicines is a pressing concern that requires immediate attention (5). The practice of irrational prescribing wastes resources and has detrimental effects for both the economy and the public’s health (6). According to estimates from the World Health Organization (WHO), almost half of all medications were improperly prescribed (7).

Observing drug use patterns in hospitals helps assess rationality, provide feedback to prescribers, and enable adjustments to maximize therapeutic benefits and minimize side effects (8). It is critical to use prescription pattern analysis to monitor prescribing practices in order to make medical care more rational and economical (5).

To the best of our knowledge, in Ethiopia, there is no study on the drug prescription pattern based on WHO drug use indicators in dermatology outpatient department (OPD). As a result, the present study aims to evaluate the prescription pattern of medications in dermatology OPD with the help of the WHO drug use indicators.

## Methods

### Study setting and period

This study was conducted from August 15 to August 30, 2024, at Injibara General Hospital, Northwest Ethiopia. Injibara, the capital of the Awi zone, is expected to have 40,836 inhabitants in 2024 (9). The city currently has one health center and two general hospitals (i.e., one public and one private hospital). Injibara General Hospital started providing dermatological services in April 2024.

### Study design

A facility-based retrospective cross-sectional study was conducted.

### Population characteristics

#### Source population

All patient prescriptions issued at the dermatology OPD of Injibara General Hospital were considered as a source population.

#### Study population

All patient prescriptions dispensed at the dermatology OPD of Injibara General Hospital from April to July 2024 constituted the study population.

#### Inclusion and exclusion criteria

All patient prescriptions issued at the dermatology OPD of Injibara General Hospital during the study period were included. Those prescriptions that were legible and complete were included for prescribing indicators. Prescriptions for patients who seek follow-up care during the study period and come back more than once were not included in subsequent visits.

#### Sample size determination and sampling procedure

The sample size for assessing prescribing indicators was based on the WHO recommendations that at least 600 encounters should be included in the survey (7). From April to July 2024, a total of 927 prescriptions that were dispensed in the hospital outpatient pharmacy were found. Out of this, 620 patients were new, and the rest were follow-up cases. Most of those patients who came for follow up received the same medication through their follow up. As a result, we included all new patient prescriptions issued at dermatology OPD of Injibara General Hospital during this time period to satisfy the WHO criteria.

#### Method of data collection

First, the principal investigator gave a half-day of training on data collection and management to a supervisor and data collectors and structured data collection tool adopted from the WHO core medicine use indicator was used to collect data. It was employed after it was translated into the Amharic version by a linguist to collect the relevant data from the patient prescription paper. The source of data was the hospital outpatient pharmacy. Before actual data collection, the supervisor and data collectors conducted a pretest at the dermatology OPD in Injibara General Hospital. A 5% of the questionnaire was tested, and 31 patient prescriptions were reviewed to complete the questionnaire. Modifications were made according to the findings of the pretest. The socio-demographic information (age and sex) was presented in the first section. The second part comprises patterns of skin diseases. The last part includes patterns of drug prescription at the dermatology OPD of Injibara General Hospital. Two trained nurses collected the data. We held discussions at the conclusion of data collecting to ensure that all the information was accurate and complete.

### Operational definitions and measurements of variables

The WHO prescribing indicators that were used in this study include (7):

- The average number of drugs prescribed per encounter: It will be calculated by dividing the total number of different drug products prescribed by the number of encounters surveyed. Combinations of drugs prescribed for one health problem will be counted as one. The WHO standard is ≤2% (1.6–1.8%).
- Percentage of drugs prescribed by generic name: It will be calculated by dividing the number of drugs prescribed by generic name by total number of drugs prescribed, multiplied by 100. The WHO standard is 100%.
- Percentage of encounters in which an antibiotic were prescribed: It will be calculated by dividing the number of patient encounters in which an antibiotic was prescribed by the total number of encounters surveyed, multiplied by 100. The WHO standard is <30% (20–26.8%).
- Percentage of encounters with an injection prescribed. It will be calculated by dividing the number of patient encounters in which an injection was prescribed by the total number of encounters surveyed, multiplied by 100. The WHO standard is <10%.
- Percentage of drugs prescribed from an Essential Drug List: Percentage will be calculated by dividing number of products prescribed that are in Ethiopian Essential Drug List by the total number of drugs prescribed, multiplied by 100. The WHO standard is 100%.

#### Data processing and analysis

Each item of data was coded and assigned a special identification number. A different value than the potential replies was assigned to the missing value. The data entry was removed if there was more than 50% of the required data missing or if a needed variable could not be obtained again. Duplicate entries were eliminated. Cross -tabulation was used to verify the answers’ logical coherence. The cleaned and edited data were ready for appropriate statistical analysis.

The data collected using a structured data collection tool adopted from the WHO core medicine use indicator was entered into Epi-data version 3.1 and exported and analyzed using Statistical Package for Social Science Studies (SPSS) version 27.1. A descriptive analysis using frequency and percentage of the variables was done. The result of the analysis was presented using text forms, tables, and bar chart.

## Results

### Socio-demographic characteristics

A total of 605 prescriptions were evaluated, yielding a response rate of 97.5%. Three hundred forty-six (57.2%) of the cases recorded were female, and the most common age group was under the age of fifteen (38.3%). Highest proportion of male patients attending the dermatology OPD were under the age of fifteen (20.3%), and in females, the maximum number of patients were in the age range of 15–29 years (21.5%). Male patients aged 60 or older made up the minimum number of patients (1.98%) (Table 1).

**Table 1:** Age with sex distribution of patients attending dermatology OPD of Injibara General Hospital, 2024.

Age (years)	Male, *n* (%)	Female, *n* (%)	Total, *n* (%)
<15	123 (20.3)	109 (18)	232 (38.3)
15–29	65 (10.7)	130 (21.5)	195 (32.2)
30–44	30 (4.95)	69 (11.4)	99 (16.4)
45–59	29 (4.79)	23 (3.8)	52 (8.6)
≥ 60	12 (1.98)	15 (2.5)	27 (4.5)
Total	259 (42.8)	346 (57.2)	605 (100)

### Pattern of skin diseases

Based on disease distribution, out of 605 patients, tinea capitis (13%) accounts the largest proportion of skin disorder, followed by atopic dermatitis (7.2%) and impetigo (6.9%) (Table 2).

**Table 2:** Commonly encountered skin diseases in dermatology OPD of Injibara General Hospital, 2024.

Disease category	Frequency (763)	Percentage
Tinea capitis	99	13%
Atopic dermatitis	55	7.2%
Impetigo	53	6.9%
Papular urticaria	39	5.1%
Seborrehic dermatitis	36	4.7%
Folliculitis/furunclosis	31	4%
Vitiligo	29	3.8%
Scabies	24	3.1%
Lichen simplex chronicus	22	2.9%
Candidiasis	22	2.9%
Tinea corporis	21	2.8%
Acne vulgaris	20	2.6%
Psoriasis	20	2.6%

### Prescribing practice

**Figure 1:**
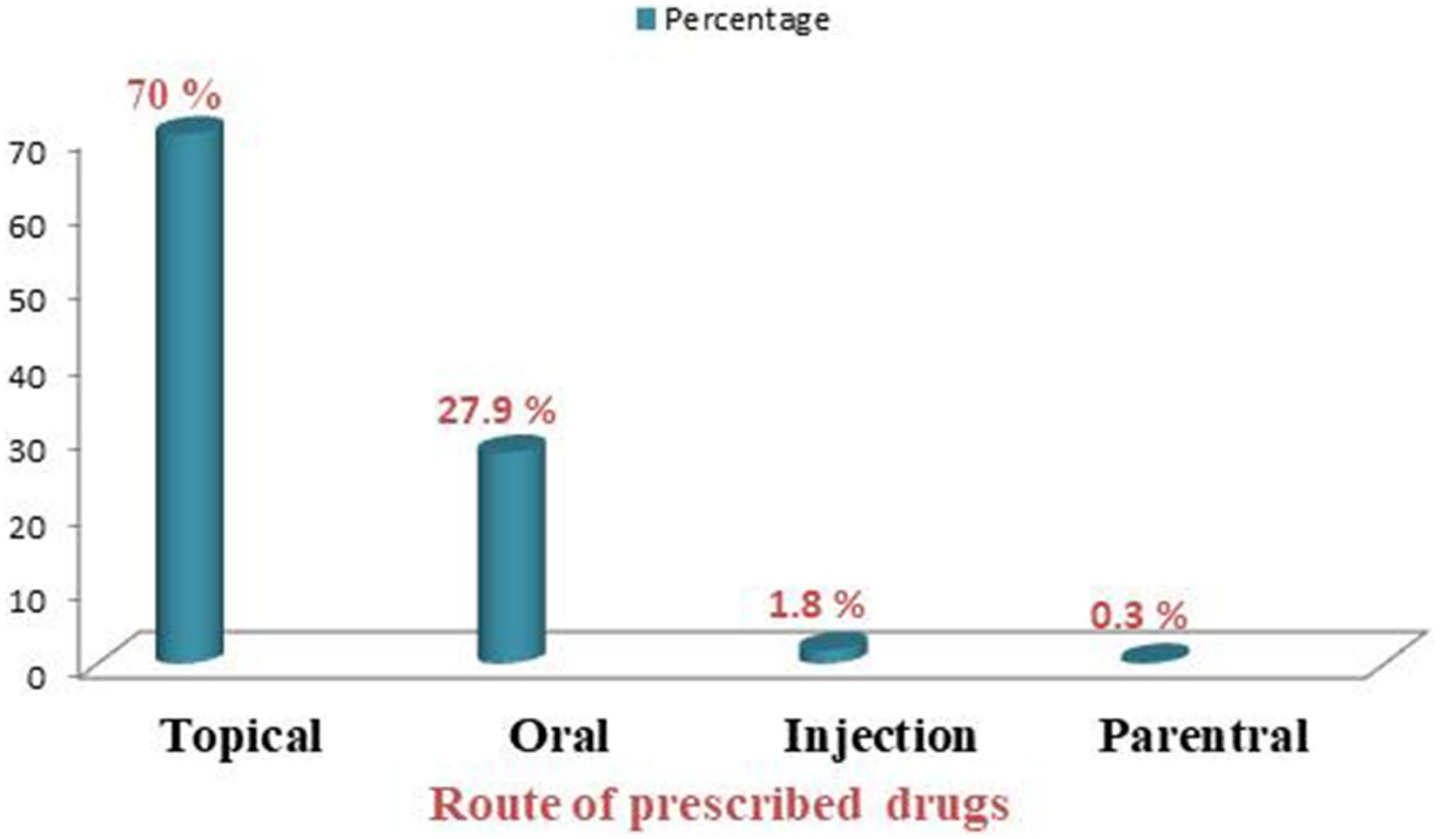
Route of administration of prescribed drugs at dermatology OPD of Injibara General Hospital, 2024.

The total number of drugs in 605 patients was found to be 1,055. Out of 1,055 drugs prescribed, topical route 739 (70%) was common route of administration, followed by oral route 294 (27.9%) (Figure 1).

The number of drugs per prescription varied from 1 to 5. Most of the prescriptions consist of a minimum of 2 drugs (422 prescriptions, 40%). The others, with 27.7 and 21.6%, contain a single drug and 3 drugs, respectively (Table 3).

**Table 3:** Number of drugs per prescription in dermatology OPD of Injibara General Hospital, 2024.

No. of drugs per prescription	No. of prescription, *n* (%)
1	292 (27.7%)
2	422 (40%)
3	228 (21.6%)
4	88 (8.3%)
5	25 (2.4%)

Topical steroids were commonly prescribed medications, accounting for 317 (30%), followed by emollients 151 (14.3%) and topical antifungals 128 (12.1%) (Table 4).

**Table 4:** Prescription pattern at dermatology OPD of Injibara General Hospital, 2024.

Drug category		No. of drugs	Percentage
Steroids	Topical steroids	317	30%
	Systemic steroids	10	0.95%
Antifungals	Topical antifungals	128	12.1%
	Systemic antifungals	119	11.3%
Antibiotics	Topical antibiotics	49	4.6%
	Systemic antibiotics	82	7.7%
Emollients		151	14.3%
Antihistamines		54	5.1%
Permethrin		24	2.2%
Topical retinoids		21	2%
Skin lightening agents		14	1.3%
Antivirals		8	0.76%
Vitamins/minerals		8	0.76%
Miscellaneous*		70	6.6%

^*^Salicylic acid, urea, ant pain, antimalarial, methotrexate, gabapentin, amitriptyline, and others.

### WHO prescribing indicators

The average number of drugs per encounter was found to be 1.74. The proportion of antibiotic prescriptions was 21.7% and the percentage of encounters with injections was 3.1%. The percentage of drugs prescribed by generic name was 95.4% and drugs from the Ethiopian essential drug list accounted for 83.6% (Table 5).

**Table 5:** Comparison of study findings with WHO standard value for drug prescription.

WHO indicators	Observed value	WHO standard value
Average number of drugs per encounter	1.74%	≤ 2% (1.6–1.8%)
Percentage of encounters with antibiotics	21.7%	<30% (20–26.8%)
Percentage of encounters with injections	3.1%	<10%
Percentage of drugs prescribed by generic name	95.4%	100%
Percentage of drugs from Ethiopian essential medicine list	83.6%	100%

## Discussion

To ensure consistent health care delivery worldwide, WHO (7) established a set of fundamental criteria that enable healthcare policy planners, managers, and researchers to compare healthcare facilities and assess health professionals’ practices toward rational drug use. These criteria include the average number of drugs per encounter, the percentage of antibiotic prescriptions, the percentage of injection encounters, the percentage of generic drug prescriptions, and the percentage of prescriptions from essential drug lists.

In the current study, the average number of drugs per encounter was 1.74, which is within the standard set by the WHO (1.6–1.8) (7). Likewise, the result of the present study was similar to a study done in Jimma University Specialized Hospital (1.7) (10). However, the result of our study was lower than the study done in selected public hospitals of Eastern Ethiopia (11), Bule Hora Hospital (12), Southern Ethiopia of eight hospitals (13), Northeast Ethiopia (14), and Borumeda (6), respectively. The average number of drugs per prescription reported in this study was also low as compared to studies from other parts of the world, including India, Nepal, Bangladesh, and Pune, respectively (1, 15, 16). The study’s optimal prescription finding can be explained by the fact that dermatological services are only provided by specialty physicians, who are less likely to use symptomatic therapeutic strategies and empirical prescribing.

The proportion of antibiotic prescriptions was 21.7%, which agrees with the WHO standard, which states that <30% of prescriptions should contain one or more antibiotics (7). This finding was lower than the finding of studies conducted in Zimbabwe (17), Jimma University Specialized Hospital (10), and the United Arab Emirates (18), Northeast Ethiopia (14), Sudan (19), selected public hospitals in eastern Ethiopia (11), Uganda (20), and in Nigeria (21, 22). Even though the prescription of antibiotics in this study was generally acceptable compared to the WHO standard and other studies, overuse and irrational use of antibiotics has become a serious potential threat, due to the rapid emergence of antimicrobial resistance and the slow pace in the discovery and development of new antibacterial agents.

This study showed that the percentage of encounters with injections was 3.1%, which complies with the WHO drug use-indicator standard (<10% of prescriptions should include injections) (7). The result of our study was lower than the study done in selected public hospitals of Eastern Ethiopia (11), Northeast Ethiopia (14), in Southern Ethiopia (eight hospitals) (13), and Bule Hora Hospital (12). This is, however, greater than the results of an Indian study, which indicated that only 0.41% of encounters involved injections (1).

In the current study, the proportion of generic drugs prescribed was found to be 95.4%, which is comparable to a study done in Bule Hora Hospital (96.8% (12)). However, it was higher than studies conducted in Northeast Ethiopia (14), selected public hospitals of eastern Ethiopia (11), Jimma University Specialized Hospital (10), Borumeda (6), and Southern Ethiopia of eight hospitals (13). This result was; however, lower than the WHO standard (100%). Even though the number is high, as the study was conducted in a public hospital, a considerable number of prescriptions were prescribed by brand, significantly causing financial wastage.

This study showed that the percentage of drugs from the essential drug list was 83.6%, which is comparable to study findings from Northeast Ethiopia (82.83%) (14). This is higher than the results from Nepal, India, and Bangladesh, which indicated that the percentage of drugs from the essential drug list was 13, 26.9, and 70%, respectively (1, 16). While prescription medication from EDL is better than previous studies, it still falls well short of WHO guidelines showing failure dermatologists to follow established guidelines.

### Strengths of the study

We used a sufficient sample size that meets WHO guidelines for evaluating drug use indicators in healthcare settings.

### Limitations of the study

Our study focused solely on prescribing indicators; out of the three pillars of WHO drug use indicators (i.e., prescribing indicators, patient-care indicators, and health-facility indicators). The non-probability sampling method we employed might not be representative of the general population. The study focuses on the prescribing practices of two dermatologists in a single healthcare setting, and hence it cannot accurately reflect the nationwide trend. Because of the retrospective nature of the study, we encountered an issue of incomplete recording of patient and drug information in some prescription papers.

### Implications of the study

1. Practice implications: The average of 1.74 drugs per encounter that is within the WHO recommendations suggests that dermatologists are likely engaging in appropriate prescribing behaviors that minimize poly-pharmacy and the associated risks. The percentage of encounters with antibiotics within the WHO recommendations reflects that dermatologists are less likely engaging in irrational antibiotic prescriptions and play a positive role in fight against antimicrobial resistance. The percentage of encounters with injection within the WHO recommendations reflects a positive aspect of prescribing practices, indicating an effort to minimize unnecessary use of injections, which can reduce the risk of infections and complications associated with injection use. The deviation of generic drug prescription from WHO standard indicates that dermatologists are less inclined to prescribe generic medications which can significantly increase healthcare costs for patients. Similarly, physicians are prescribing fewer medications from the Ethiopian essential medicine list, which reflects lack of adherence to established guidelines.
2. Policy implications: The positive finding related with the average number of drugs per encounter and percentage of encounters dispensing antibiotics and injections can inform policymakers about the current state of prescription indicators in dermatology outpatient department and help them to further promote the best practices. On the other hand, the low prescription of generic drugs and medications from Ethiopian essential drug list suggest a need for policies that promote the use of essential medicines and generics, ensuring that patients have access to affordable treatments while maintaining quality care.
3. Research implications: The study findings can be used as a baseline data for the future researchers.

## Conclusion and recommendations

The average number of drugs per encounter and percentage of encounters dispensing antibiotics and injections were within the WHO recommendations. The percentage of drugs prescribed by generic names and the percentage of drugs from the Ethiopian essential drug list were below the WHO standard value.

It is recommended that dermatologists need to improve, especially when it comes to prescribing generic drugs and medications from the Ethiopian Essential Medicine List. The low percentage of drugs prescribed from the Ethiopian essential drug list indicates a need for greater adherence of dermatologists to established guidelines.

Policymakers should consider developing strategies to promote the use of the essential drug list and generic drugs among dermatologists, possibly through robust monitoring and evaluation systems. Encouraging prescribers to familiarize themselves with and utilize the essential drug list can ensure that patients receive appropriate and cost-effective treatments. Promoting the use of generic names in prescriptions can significantly reduce healthcare costs for patients. This is particularly important in resource-limited settings where patients may struggle to afford brand-name medications.

Future researchers are encouraged to conduct a large-scale study to understand the national drug prescribing pattern in dermatology OPD in accordance with WHO standards, taking into account WHO patient-care indicators and health-facility indicators. This is because the study solely looks at the WHO prescribing indicators and the prescribing practices of two dermatologists. The study used the WHO prescribing indicators, which are supposed to record exactly what is prescribed to patients, but not why. As a result, the study’s findings emphasize the necessity for future studies to investigate why dermatologists prescribe less of generic medications and those on the Ethiopian Essential Medicine List.

## Data Availability

The original contributions presented in the study are included in the article/supplementary material, further inquiries can be directed to the corresponding author.
